# Bio-Stimulated Lower Limb Rehabilitation Robot Semantic Analogy Fit Design

**DOI:** 10.3390/biomimetics10030134

**Published:** 2025-02-24

**Authors:** Tianyi Yao, Hongfei Yu, Zhongzhi Qin, Li Sun, Jiantao Wu

**Affiliations:** 1 School of Art and Design, Yanshan University, Qinhuangdao 066000, China; yaotianyi@stumail.ysu.edu.cn (T.Y.); qinzhongzhi@ysu.edu.cn (Z.Q.); wjt@stumail.ysu.edu.cn (J.W.); 2 School of Mechanical Engineering, Yanshan University, Qinhuangdao 066000, China; sunli@ysu.edu.cn; 3 Hebei Technology lnnovation Center for Intelligent Industrial Design, Qinhuangdao 066000, China

**Keywords:** biological incentives, lower limb rehabilitation, analogy analysis, similarity calculations, bionic design

## Abstract

In order to solve the problem of insufficient design applicability in the field of lower limb rehabilitation, such as interaction, experience comfort, and modeling color, a biological excitation function system was used to guide the solution of the functional scheme of lower limb rehabilitation products, and the transformation of lower limb rehabilitation products in functional interaction, experience, and morphological color design driven by biological information-driven cross-domain mapping was improved. We used patent knowledge mining to study the product functional requirements of lower limb rehabilitation products. The results were used to screen the required biological prototypes, and the biological incentives were used to guide the design problems. According to the principle of analogy and similarity calculation, the similarity matrix was obtained, and then the strategy was analyzed. Through the analogy of functional system–product technology engineering systems, the engineering relationship between multi-biological and multi-design elements was determined. We realized the biological replacement and upgrading of product functions under biological stimulation to guide the design of lower limb rehabilitation products. The accurate quantitative biological information of multi-biological analogy fit has the significance of optimizing the training effect, improving the operation efficiency, and improving the morphology and modeling of the lower limb rehabilitation product engineering transformation and design. The acquisition rate of the functional design requirements of lower limb rehabilitation products based on text mining reached 95%, and the accuracy of the biological design prototype obtained through similarity calculation was higher than 79%, which verified the feasibility of the accurate bioinformatics design method and improved the rigor of the bioinformatics biomimetic design method.

## 1. Introduction

As an important branch of rehabilitation aids, lower limb rehabilitation has become an important auxiliary device for lower limb rehabilitation training for stroke patients. Currently, the disability rate is higher than 80%, and the severe disability rate is higher than 40%. Regardless of the functionality of the product and the needs of patients and medical staff, it is necessary for researchers to design more efficient and applicable lower limb rehabilitation products. Vandevenne [1] measured the influence of Ask Nature on the creative results of biomimetic design through comparative experiments, and the results showed that its functional scope could conceive more different design concepts at the abstract level. Jon-Michael Deldin [2] elaborated on the strategy of using the Ask Nature biological knowledge base, and pointed out the commonality of functions for biomimetic classification and product requirement definitions as a further research direction. Liu Wei, Cao Xiaoliang, Hou Xiaoting et al. [3,4,5] provided biological modeling methods by exploring the information and knowledge of multi-biological effects, and analyzed the development direction and functional combination of biological knowledge and engineering technology of biomimetic design. Wang Huan et al. [6] expanded the application scope of multi-biological effects, combined them with aesthetics, and formed design methods and processes from the perspective of product emotion, which made up for the shortcomings of previous applications of multi-biological effects in product design. Wu Xubo et al. [7] constructed a research system for biomechanics, using theoretical knowledge of sports injuries, motion capture, and clothing material testing experiments for protective sportswear product development. Shi Dongyan, Tan Runhua and other scholars [8,9] pointed out that the types of effects can be divided into physical, chemical, and geometric effects according to the relationship between input and output. During the long process of natural selection, after hundreds of millions of years of evolution and elimination, biological systems have formed their own distinctive functions and morphological structures, adapted to the changing environment, which solved the problem of survival. The preserved biological systems are life-cycle sustainable [10,11], capable of efficient use of energy [12,13], self-controlled through learning and feedback, and self-assembled using synthesis and replication. Biological systems exhibit significant advantages over man-made systems in many ways [14], and these superior properties are unmatched by man-made technical systems. With the development of natural sciences and the deepening of biological research, large-scale biological phenomena, biological examples, and other biological information have been presented to people [15]. According to statistics, about one million new biological research results are published each year [16]. Usually, this biological information contains a large amount of design knowledge with engineering application value, such as function, principle, structure, behavior, strategy, etc., which can provide effective solutions for complex design problems, can be fully applied to product concept design, and help to realize product innovation. Biomimicry generally refers to the technical science that studies the principles, structures, morphology, behaviors, and interactions of biological systems in order to provide new operating principles and system compositions for engineering technology [17]. As a comprehensive interdisciplinary discipline, it is the result of the interpenetration and combination of life sciences and engineering technologies [18]. It focuses on scientific discovery, which is an important way to explore, recognize, understand, and generate new knowledge [19], and is the theoretical basis for human beings to use biological systems for technological innovation, providing opportunities for the discovery of new knowledge and knowledge transfer between fields.

Problems faced by human society are solved by selecting excellent examples from nature and mimicking the principles, structures, and processes of living organisms [20]. We can apply the innovative inspiration obtained from nature to human society [21,22], generating new ideas and solutions that can solve human problems, and putting them into practice. Bio-inspired design is a knowledge-driven design approach [23], which uses biological domain knowledge to provide inspiration for problem solving and technological innovation in the engineering field through systematic cross-domain knowledge retrieval and analogy. Emphasizing the systematic nature of the design approach, the focus is on transferring knowledge from the biological domain to the cross-domain of engineering from a more novel and comprehensive perspective. Inspired by biological knowledge [24], the goal of biostimulus design is not to replicate biological features exactly but to extract basic principles and apply them to the engineering field to generate new solutions [25,26].

Biomimetic design and biostimulus design require collaboration between biology and other disciplines [27,28], involving biologists, physicists, chemists, and materials scientists understanding biological principles, structures, and functions [29], as well as engineers’ guidance on the design of various materials or devices. This interdisciplinary collaboration studies the excellent properties of biological systems and uses them as reference models, using technical means to reconstruct product objects and working processes [30], so as to achieve the purpose of applying biological characteristics to technical systems. Both biomimetic and biostimulus design involve special forms of analogy [31], which are long-distance analogies from the biological “source domain” to the engineering “target domain”, which can obtain non-traditional solutions to problems and are often more innovative [32]. In addition, they are both effective means of product innovation [33,34], and the operating mechanism of biological systems is different from that of technical systems, and the application of the principles, structures, and related biological knowledge refined in the biological field can provide a unique perspective for improving existing equipment and designing new products, which can help generate new solutions and trigger disruptive innovation [35].

In recent years, scholars at home and abroad have tried to apply different methods to construct the cross-domain mapping of information between engineering and biological fields. Chiu et al. [36] from the University of Toronto used synonyms of engineering terms in WordNet to establish a mapping to bioinformatics. WordNet is a database of English vocabulary developed based on psycholinguistics [37], which is mainly composed of nouns, verbs, adjectives, and adverbs, and establishes connections between words through semantic networks. Similarly, Shu et al. [38] proposed the use of engineering terms to establish biometric mapping in VerbNet. VerbNet is an English verb database developed based on Levin’s verb classification standard [39], which contains grammatical and semantic information of verbs. Lenau et al. [40] first performed a preliminary search in a specified database (e.g., AskNature, Britannica Online) using engineering terminology, and then used the biological terms contained in the search results to construct cross-domain mappings. Professor Vincent of Heriot Watt University in the United Kingdom conducted a comparative analysis of engineering systems and biological systems, and found that the essential difference between the two is that engineering tends to use energy and materials to achieve the same purpose, while living organisms use more information and structures [41]. For example, traditional buildings use air conditioning systems to regulate temperature at the cost of electrical energy, while Zimbabwean termite burrows use radial ventilation structures that are perpendicular to each other to achieve the same purpose. A. Brahmia and R. Kelaiaia analyzed the physiological research results of knee joints in different motion states and the friction coefficient of the articular surface during the training of healthcare-assistive devices, so as to provide a theoretical basis for the design of rehabilitation training mechanisms.

In the current field of research on lower limb rehabilitation robots, traditional bionic tools reveal significant deficiencies. They exhibit shortcomings in the mapping of biological and engineering functions, resulting in a substantial reduction in similarity when accurately mapping biological prototypes to engineering designs, which directly negatively impacts the design quality of rehabilitation products. Furthermore, existing research lacks depth in exploring the adaptability of lower limb rehabilitation robots in different rehabilitation environments, as well as how to facilitate good interaction with patients and healthcare personnel. There is also insufficient research on the impact of design semantics on patients’ rehabilitation motivation and compliance. Additionally, the bionic design research of lower limb rehabilitation robots based on biological information incentives has yet to propose practical and effective countermeasures and solutions. Currently, most studies on biological effect incentives primarily focus on the theoretical improvement of design methods, with very few tangible results in practical design applications. Within the scope of applied research on lower limb rehabilitation robots, the focus has largely been on mechanical equipment mechanisms, performance, and materials, while the application of bionic design under biological effect incentives is extremely scarce. At present, most lower limb rehabilitation research relying on bionic design exhibits considerable subjectivity, often depending on a single biological prototype for localized design, leading to a lack of systematic and comprehensive transformation in product bionics.

In view of this, this study proposes an innovative design approach. In this method, biomimetic objects are screened by quantitative and objective means, and the biological incentive system is used to guide the design process. Through patent knowledge mining, in-depth analysis of product functional requirements, product function decomposition, construction of a function tree model, and statistical judgment of functional demand frequency, based on the use of the results to accurately screen the biological prototypes in the required biological knowledge base Ask Nature, biological incentives to guide the design of problems, according to the principle of analogy analysis of biological functional systems and technical engineering systems, through similarity calculation and obtaining a similarity matrix, were screened to obtain accurate and effective biological functional prototypes and analyze their strategies. Through the analogy of the functional system of biological excitation and the technical engineering system of the product, the engineering relationship transformation of multi-organism to multi-design elements is constructed. We realize the biological replacement and upgrading of product functions under biological stimulation to guide the design of lower limb rehabilitation products. Different from traditional bionic design, which only focuses on the shape or structure of single-level biomimicry, this study uses the principle of analogy to deeply analyze the biological functional system and the technical engineering system from multiple levels of morphology, behavior, movement, ecology, etc., calculates the similarity between the two, and establishes the mapping relationship between the multi-level correspondence. This innovative design method not only enriches the source of bionic creativity of lower limb rehabilitation products but also effectively improves the emotional value and user satisfaction of the products, provides a more accurate and systematic functional optimization solution for the design of lower limb rehabilitation products, fills the gap in existing research, and opens up a new direction for the development of this field.

## 2. Requirement Extraction and Reasoning Methods

### 2.1. Patent Knowledge Mining of Functional Requirements

#### 2.1.1. Patent Knowledge Mining

**Figure 1:**
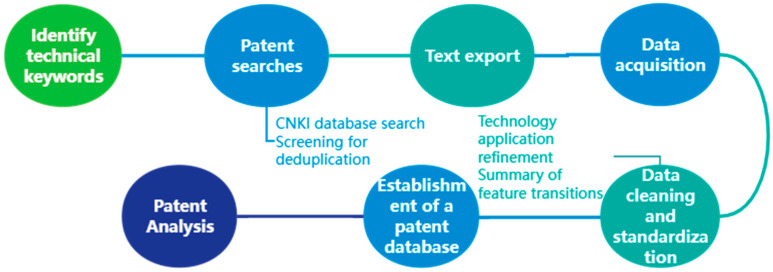
Flow chart of patent knowledge acquisition.

Patent knowledge mining is the mining of patent data, and the process of retrieving the knowledge hidden in the special relationship from a large number of patents. The characteristics of the product structure, form, etc., constitute the innovative principle of the patent. Through the methods of text mining, information extraction, distribution prediction and others, the hidden, potentially useful knowledge is extracted from the patent literature information (patent title, abstract, claims, and description). The transformation and migration of technologies in this field are discovered, the correlation between key elements of patents is revealed, and a knowledge base of modifiable design is obtained for the field of lower limb rehabilitation robots by using patent information mining. The process of obtaining patent knowledge consists of the following steps, as shown in Figure 1.

#### 2.1.2. Product Demand Extraction

According to the keywords of the lower limb rehabilitation product and the design carrier used to collect the patent information, through the technical application provided by the patent text information and the introduction of the patent specification, the functional transformation results of each lower limb rehabilitation robot patent are summarized from the principles of redundant lower limb rehabilitation product technology and machinery. Table 1 shows some of the functional conversion elements, and the rest is shown in Appendix A [42,43,44].

**Figure 2:**
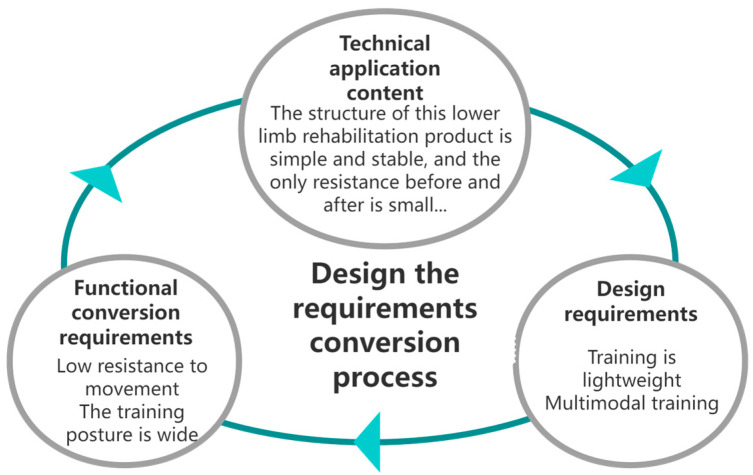
The process of refining and converting requirements.

Starting from the content of the patent, through patent analysis and combined with reality, a large amount of patent knowledge related to technological development and functional evolution can be obtained. Through this method, the functional description text corresponding to each product can be studied in more depth. The extraction method of multi-stage group screening achieves the transformation of the technology mentioned in the patent into functional requirements, and functional requirements guide design requirements. Figure 2 shows the obtained design requirements, which can be used as a complete corpus collection.

**Figure 3:**
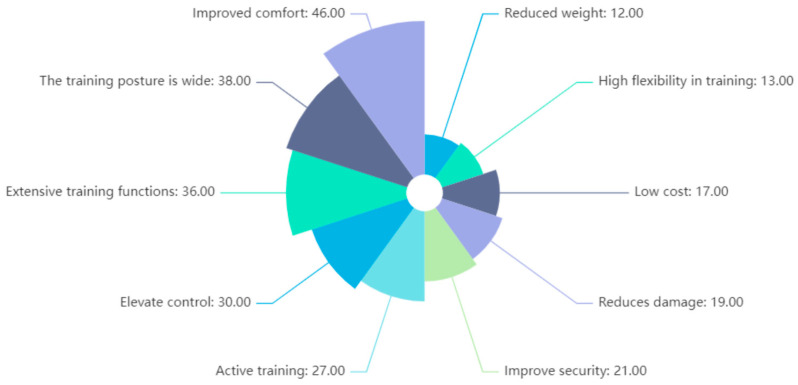
Demand frequency statistics histogram.

Then, for about 259 patented products under the keyword of lower limb rehabilitation products, text mining of the patent specification was carried out, and similar functions of the basic information of the patent were integrated and recorded. According to the corpus collection of lower limb rehabilitation products made by patent knowledge mining in the earlier part, the data collection determined the weight of the problem for the product demand function, so frequency statistics calculations of the lower limb rehabilitation products were carried out to create a histogram, as shown in Figure 3.

**Figure 4:**
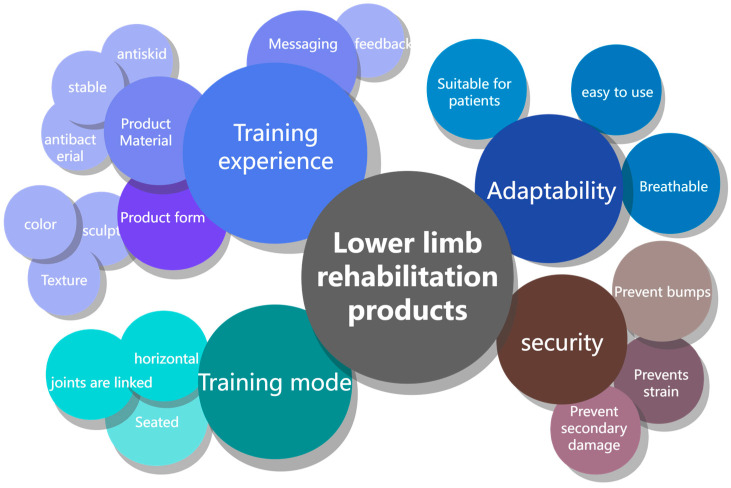
Functional tree model.

Under the conditions of the above-mentioned functional requirements of the product, it is also necessary to perform a functional analysis of the product for biostimulus transformation. The purpose of functional analysis is to determine the design goals. Just like the extraction principle of the biological effect model introduced above, the design requirements are taken as the “input” content, the “output” result is the desired product function, the middle preset part is all the functions that are converted into outputs, and finally, the content of the target product is the interrelationship of the target product. The final functional tree model is shown in Figure 4.

### 2.2. Analogy Source Reasoning and Similarity Calculation

**Figure 5:**
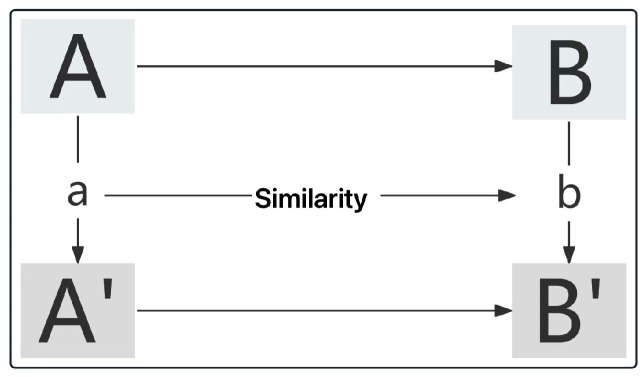
Analogy reasoning flow.

The purpose of analogical source reasoning is to find two systems, process the source reasoning of one system through the other, quantitatively calculate the degree of similarity, and judge the reasonableness. In the analogy process, the initial period of the biological prototype can be A, the product carrier is B, and the biological incentive at the end is A’ and the product output is B’, as shown in Figure 5.

It is known that the biological excitation system *A* contains {*a*_1_, *a*_2_, *a*_3_,…, *a*_n_}, and product system *B* contains {*b*_1_, *b*_2_, *b*_3_,…, b_m_}, namely

*A* = {*a*_1_, *a*_2_, *a*_3_,_…_, *a_n_*}

*B* = {*b*_1_, *b*_2_, *b*_3_,*_…_*, *b_m_*}

As can be seen from the above, the two systems exhibit a parallel evolutionary process, so in order to maintain the close connection of the steps, a set *Z* represents the similarity, and the set element is *U*,*U* = {*u*_1_, *u*_2_, *u*_3_,…, *u*_z_}.

We set *S*(*A*,*B*) to indicate the degree of tightness between systems; the value range is 0–1, and the degree of compactness is positively correlated with it. The larger the value, the higher the degree of compactness, and the calculation formula is as follows:

(1) $$\begin{array}{ll} S_{\left(A,B\right)} & =\frac{Z}{N+M-Z}\sum_{i=1}^{Z}\omega_{1}s(u_{1})+\omega_{2}s(u_{2})+\cdots +\omega_{Z}s(u_{Z})\\ & =\frac{Z}{N+M-Z}\sum_{i=1}^{Z}\omega_{1}s(u_{1}) \end{array}$$

In Equation (1), ω table weight coefficient takes the value (0, 1); therefore, $\sum_{i=1}^{Z}\omega_{i}=1$; *q*(*u*_i_) is the closeness of similar elements. Knowing that the similar set between *A* and *B* is *U* = {*u*_1_, *u*_2_, *u*_3_,⋯, *u*_z_}, now let *u*_i_ and *u*_j_ in *u*_ij_ express the importance of related elements, so the importance matrix *W* can be expressed as

(2) $$W=\left[\begin{array}{cccccc} u_{11} & u_{12} & \cdots & u_{1j} & \cdots & u_{1z}\\ u_{21} & u_{22} & \cdots & u_{2j} & \cdots & u_{2z}\\ \vdots & \vdots & & \vdots & & \vdots \\ u_{i1} & u_{i2} & \cdots & u_{ij} & \cdots & u_{iz}\\ \vdots & \vdots & & \vdots & & \vdots \\ u_{l1} & u_{l2} & \cdots & u_{lj} & \cdots & u_{lz} \end{array}\right]$$

In the matrix *W*, *u_ii_* = 1, $u_{ij}=\frac{1}{u_{ji}}$*u_ij_ =* (1, 2, …, 10), the lower the value, the lower the correlation between *u_i_* and *u_j_*. Next, the maximum eigenvalues λ_max_ of the matrix W and the corresponding eigenvector *V* = {*x*_1_, *x*_2_, *x*_3_,⋯, *x*_n_} are solved, and the eigenvectors are normalized to obtain a vector set representing the weights of similar elements $\omega =\left\{\omega_{1}\right.,\omega_{2},\omega_{3}\cdots \left.\omega_{i}\right\}$.

From the above, it can be seen that the biological incentive system and the product system have been set as *A*, *B* in a set of two elements, and the selection method is based on the similarity characteristics. We set the similarity closeness of the *z*-th similar feature in the *i*-th similarity feature to $s_{i1},s_{i2},\cdots s_{ij,}\cdots s_{iz}$. Table 2 shows the value range of similarity closeness. Values need to be assigned to different feature weights to establish $d_{1},d_{2},\cdots ,d_{z}$.

The similarity element is calculated as follows:

(3) $$s(u_{i})=\frac{z}{n+m-z}\sum_{j=1}^{z}d_{j}s_{ij}$$

The value range of *dj* is 0–1; moreover, $\sum_{j=1}^{z}d_{j}=1$. In Equation (1), $\frac{Z}{N+M-Z}$ is the influence of the similarity factor between the two systems on the similarity closeness of the similar quantity *Z*; $\omega_{z}q(u_{z})$ is the effect of the weight of the similarity element on the closeness of similarity. Therefore, the more closeness elements in the two systems, the higher the correlation between the systems.

## 3. Results

### 3.1. Biological Incentive System–Product Model and Transformation

#### 3.1.1. Derivation of Analogous Models Between Systems

**Figure 6:**
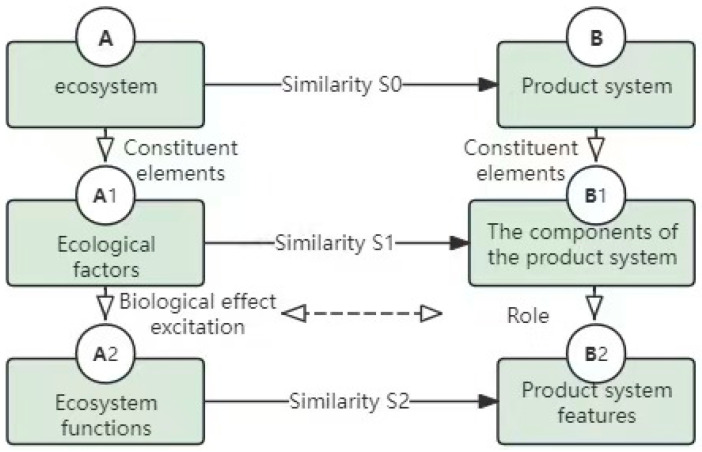
Analogy of the multi-level transformation model between the two systems.

The similarity of the analogous elements in the biological functional system and the product technology engineering system directly leads to the accuracy of the design output, which is the key issue in the design process of this method. The capture of biological prototypes and their effects, through similarity analogy analysis, can accurately obtain the morphological structure, motor function, and multi-level mapping of the living environment of biological prototypes, which is useful for the design of lower limb rehabilitation products by constructing biomimetic sources. Figure 6 shows a detailed analogy multi-level transformation model between the two models, using multi-level biological effects to guide lower limb rehabilitation products, such as redundant morphological structure, single movement mode, and asympathy to adapt to scenarios.

#### 3.1.2. Engineering Transformation of Multi-Biological Effect Prototypes

In the process of similarity analogy analysis, many biological prototypes meet the needs of the product functional system at least step by step, and the fuzzy mathematical similarity evaluation is used to quantify the compactness of the final prototype in the circle with almost the same similarity tightness. The similarity is *S*, and the similarity element is the morphological structure, motor function, and living environment factors corresponding to multiple organisms, which correspond to five biological prototypes, so the similar set of bamboo fibers is *U*_1_

*U*_1_ = {*u*_1_*, u*_2_*, u*_3_} = {Morphological structure, motor function, living environment} (4)

Therefore, the values of *N*, *M*, and *Z* are 3, and can be seen in Equation (1).

(5) $$\begin{array}{ll} S & =\frac{Z}{N+M-Z}\sum_{i=1}^{Z}\omega_{1}s(u_{1})+\omega_{2}s(u_{2})+\cdots +\omega_{Z}s(u_{Z})\\ & =\frac{Z}{N+M-Z}\sum_{i=1}^{Z}\omega_{1}s(u_{1}) \end{array}$$

The weight value ω of each similar element set *U*_1_ is taken, and the relative weight value judgment matrix is established according to the judgment matrix of Equation (2).

(6) $$W=\left[\begin{array}{ccc} 1 & 5 & 5\\ 1/5 & 1 & 1\\ 1/5 & 1 & 1 \end{array}\right]$$

According to the calculation, the maximum eigenvalue is *λmax* = 3, the eigenvectors are *V* = {0.973 3, 0.193 5, 0.192 5}, and the value of the normalized weight ω = {0.721 2, 0.141 9, 0.142 9}.

In Equation (3), the values of *n*, *m*, *z*, and *d_j_* are 1, which can be calculated according to the following equation:

(7) $$s(u_{i})=s_{ij}$$

According to the similarity tightness of the two systems of bamboo fiber and lower limb rehabilitation products, the element similarity *s* = {0.8, 0.8, 0.6} can be obtained according to the following formula:

*S* = 0.721 2 × 0.8 + 0.141 9 × 0.8 + 0.142 9 × 0.6 = 0.77622 (8)

Through the above calculation process, the similarity of the other four biological objects was evaluated and calculated, and for the other biological prototypes with similar similarity except for the selected five items, the structural characteristics of shark skin were compared with the lotus leaves, and the quantitative value of the similarity of lotus leaves was higher according to systematic calculation.

After determining the bionic object of specific biological incentive guidance, the reference entry coding and database construction were carried out through the biomimetic classification biological function system of nature to facilitate the later classification to obtain effective information and collect the corpus of biological effects through the coding library. In order to obtain accurate biological strategy information, we performed analogous analysis and screening so as to obtain the best matching degree in a certain function. In the process of screening, it is necessary to abide by the four principles of biological effects: green design, high efficiency, morphological matching, and technical feasibility. Table 3 below shows the specific targets of biological incentives.

**Figure 7:**
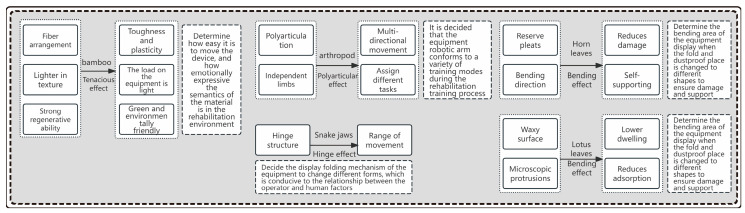
Transition route under multi-biological excitation effect.

In the engineering transformation problem of multi-biological feature visualization under biological excitation, the characteristics of the organism itself and the biological incentive solution for the design of lower limb rehabilitation products are demonstrated through the comparison of five effect transfers, as shown in Figure 7, in which the tenacity effect characteristics of bamboo and its own light texture and strong renewability are empowered by the transformation of part of the outer casing of the lower limb rehabilitation products, which have the excellent characteristics of reducing the weight of the equipment and being green and environmentally friendly.

**Figure 8:**
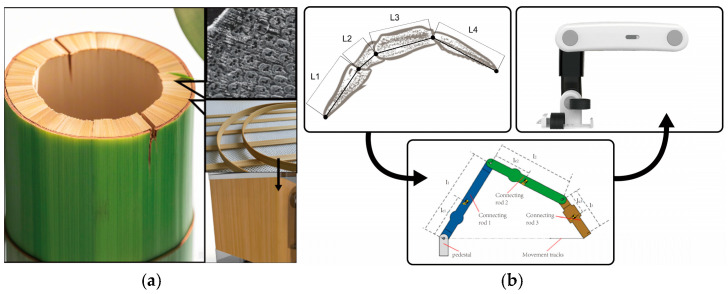
(**a**) Bamboo fiber effect inspires product shell. (**b**) Multi-joint effect inspires robotic arm.

Bamboo is arranged vertically by vascular bundles encased in cellulose fibers, embedded in an amorphous matrix, resulting in a tough effect. Bamboo winding technology is used in many fields by using bamboo as the base material and resin as the adhesive for winding processing technology, such as bamboo-based composite materials, which have the characteristics of earthquake resistance, settlement resistance, thermal insulation and anti-freezing, corrosion resistance, environmental protection, and being green and lightweight. The bamboo material process can be used in the modeling design research of lower limb rehabilitation products, which can achieve lightweight, tough, durable, and green products, as shown in Figure 8a.

Lower limb rehabilitation products serve patients with lower limb paralysis caused by stroke and other injuries; using the limb joint characteristics of arthropods, each trunk of the limb cooperates with each other to complete circular movements under a degree of non-freedom, and the lower limb rehabilitation robot mimics their movement trajectory characteristics to help the patient passively complete movements of the hip joint, thigh, knee joint, calf, ankle, and foot. The main mechanism of the robotic arm is shown in Figure 8b.

**Figure 9:**
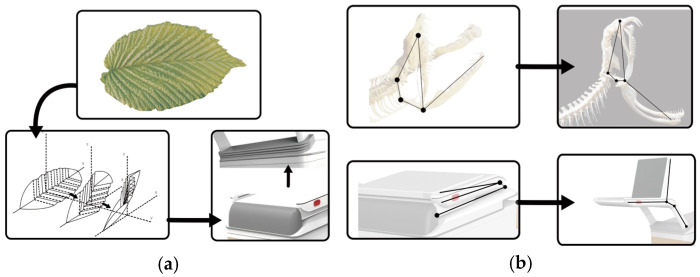
(**a**) Elm leaf folding effect inspires the dust guard; (**b**) snake jaw hinge effect inspires the operating end.

Elm leaves make use of simple folds that emanate from both sides of the central leaf vein and are repeated along the length of the leaf, with the folds of the trough leaving room for the leaves to bend inward when needed, and the crown folds keep the leaves in a rigid shape when photosynthesis requires them. Overall, this corrugated pattern allows the blade to self-support without compromising flexibility. When the lower limb rehabilitation robot changes into different forms, flexible materials are used to preserve the bending area to ensure that the service life is increased and the product damage is reduced, as shown in Figure 9a.

The snake jaw bone structure is connected to the skull by a hinge structure, with a larger open caliber. The left and right sides are connected to the ligaments, and through the ligament stretching, the width of the caliber is enhanced, and the skin with high flexibility and strength can be used to swallow prey food that is larger than its body, as shown in Figure 9b.

**Figure 10:**
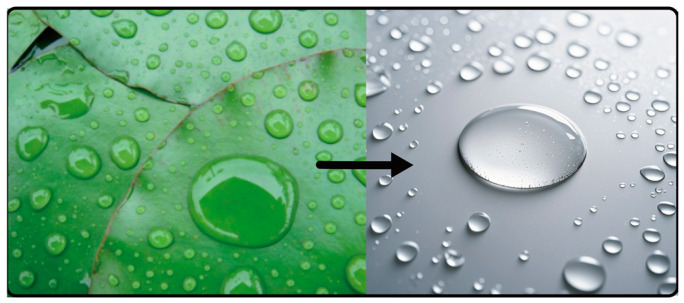
The self-cleaning effect of lotus leaves inspires the product coating materials.

Lotus plants remain dust-free, which is a distinct advantage for aquatic plants that live in muddy habitats. The waxy surface, as well as the presence of microscopic protrusions, prevents water molecules from attaching to the surface. Instead, the water will roll down, carrying away any dirt or oil on the surface of the lotus leaf along the way. Inspired by the self-cleaning mechanism of lotus plants and other organisms, a similar design was made on the external body surface of the lower limb rehabilitation product, which is conducive to maintaining the cleanliness of the product, as shown in Figure 10.

Through the identification and analysis of the above five biological prototypes, the improvement of the semantics of the prototype design of the biostimulation system of the lower limb rehabilitation robot is summarized as shown in Table 4.

#### 3.1.3. Biological Analogy Fit Product Scheme Design

**Figure 11:**
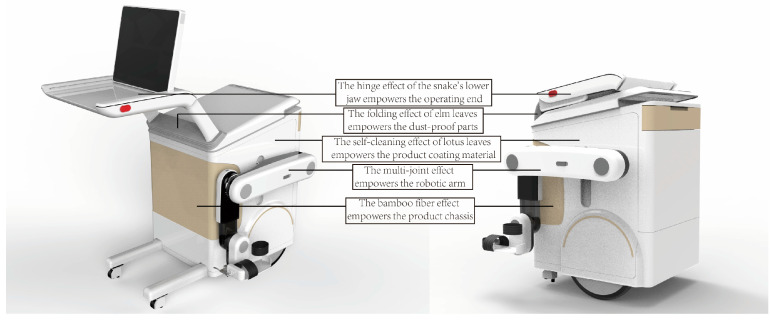
Product solutions for multi-biological analogy.

**Figure 12:**
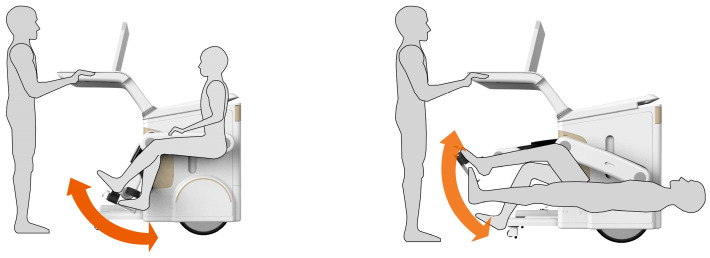
Simulation of the use scenario of multi-biological analogy fit products.

As shown in Figure 11 and Figure 12, through the analysis of human gait, the lower limb rehabilitation product is optimized for the process of landing one side of the lower limb, raising the foot, and landing it again, wherein the position space is divided into support and swing, similar to a three-link mechanism movement, which is the situation for medical staff operation and patient training processes.

In terms of morphological structure and motor function, the product uses the bone structure of the snake’s jaw to store the operating platform used by medical staff and has reasonable adaptability from the perspective of operation. The arthropod leg movement principle diversifies the mode of rehabilitation training for patients, and the lightweight and toughness characteristics of bamboo fiber improve the weight of the product. In terms of the living environment, the folding characteristics of the horn leaf structure solve the problem of prolonging the service life of the wear-prone parts of the product, and finally, the dirt that will be encountered during the use of the product can be cleaned using the biological characteristics of the lotus leaf.

## 4. Discussion and Conclusions

In this paper, a lower limb rehabilitation robot based on biological effects is innovatively designed and studied by using multiple biological effect strategies through the basic research of functional design principles and biological effects. The product demand analysis and design research method are applied using an interrelated framework. Firstly, literature mining was used to clarify the direction for the analysis of biological effect strategies. Secondly, the key elements of the biological incentive system and the product system were accurately collected and quantified by taking the similarity and closeness of the redundant and large number of biological effect strategies as the core, and the solutions suitable for meeting the needs of lower limb rehabilitation robots were screened from the same biological effect strategies with solution results. Finally, under the premise of conforming to the basic elements of the design, the morphological and functional design of the lower limb rehabilitation robot was carried out. The use of biological effects to design lower limb rehabilitation robots has improved the rehabilitation function of the product to a certain extent, and the lower limb rehabilitation robot has the characteristics of green environmental protection, perfect functions, and safety training by relying on the wisdom bred by nature.

### 4.1. Key Objectives and Outcomes

By using the method of patent knowledge mining to explore the design needs of lower limb rehabilitation robots, the concerns and frequency of user needs in the field of lower limb rehabilitation robots in enterprises, schools, and research institutions were accurately judged based on patent knowledge. Then, a functional tree model of the lower limb rehabilitation robot was created.

Based on the substitution relationship between biological principles and engineering technology principles, this paper proposes the functional design of lower limb rehabilitation robot products based on biological effects, the analysis of biological functional systems and technical engineering systems based on the principle of analogy, the similarity calculation and the similarity matrix, the screening and acquisition of accurate and effective biological functional prototypes, and the analysis of their strategies, engineering technology substitution, and design schemes. In the engineering substitution part, the optimal biological effect strategy for the needs of lower limb rehabilitation robots was clarified.

### 4.2. Conclusions and Prospects

Patent knowledge mining of lower limb rehabilitation robot information can effectively collect policy information. The main source of information in the process of technological innovation comes from patent data. Patent information contains 90–95% of the world’s research results. In enterprises and research institutions, the effective use of patent information can save nearly 40% of the research and development costs, and shorten the research and development time by 60%. The patent acquisition method is extensively used in the design and research of the lower limb rehabilitation robot, and the functional requirements of the lower limb rehabilitation robot and its patent knowledge are accurately integrated. This method also provides a real, effective, fast, and convenient basis for the problem mining of special research in the field of healthcare in future research.

Against the social background that lower limb rehabilitation robots are an important part of the rehabilitation field, this paper takes stroke and lower limb paralysis patients and medical staff as the service objects, guides the design through the innovative integration of biological effects into a lower limb rehabilitation robot, and designs a lower limb rehabilitation robot that optimizes the rehabilitation training effect, increases the operation efficiency, and improves the shape, so as to ensure that it can complete the rehabilitation training work efficiently for various physical and mental health conditions.

The design and research of the lower limb rehabilitation robot are attached to the prototype construction test project of the lower limb rehabilitation robot of the School of Mechanical Engineering, and the linear rehabilitation training experimental test, the stepping circle rehabilitation training test, and the patient fatigue test are being gradually improved, but they are still in the experimental stage, and it is not convenient to disclose them at present. Based on the semantic analogy fit design of the bio-stimulated lower limb rehabilitation robot, product development is feasible in the application, and the training displays stable interactive operation in the experimental test process, which confirms the correctness of the design for the research and development of the lower limb rehabilitation robot.

## Figures and Tables

**Table 1:** Some elements of patent knowledge mining function transformation (patents from CNKI Patent Database).

Patent Publication Number	Patent Name	Application	Functional Conversion
CN112451320A	Weight-loss support mechanism and lower limb rehabilitation robot including it	Bracket assembly, support, rope assembly	Reduced resistance and a wide range of training postures
CN109009885B	An easy-to-use exoskeleton-type lower limb rehabilitation robot	Drives, movable devices	Adjustable height
CN112386871A	A rehabilitation robot for gait rehabilitation training of lower limbs that is convenient for moving	Motors, threaded shafts	Adjustable auxiliary structure

**Table 2:** The values.

*S_ij_*	Meaning
0~0.2	The two features are extremely closely related
0.2~0.4	The two features are less closely related
0.4~0.6	The two features are moderately close
0.6~0.8	The two features are more closely related
0.8~1.0	The two features are extremely closely related

**Table 3:** Specific objects of biological incentives.

Name of the Effect	Presentation Diagram	Model Analysis
Bamboo fiber effect	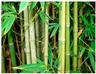	The cross-section of the tube wall shows that the vascular bundles are arranged in a density gradient. On the inner surface of the tube wall, the bamboo tissue is mainly the matrix, while the proportion of strong vascular bundles increases towards the outer edge
Arthropod effect	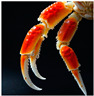	Arthropod limbs allow for multiple degrees of freedom of movement by clustering two or three joints in one limb
Elm leaf folding effect	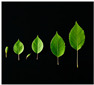	The folds leave room for the leaves to bend inward when needed. When required for photosynthesis, the crown folds keep the leaves in a hard shape
Snake jaw hinge effect	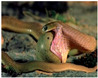	The bones on each side of the snake’s jaw are joined together by three hinge points, which promote the flexible epidermis to which the bones are attached by the force of the muscles
Self-cleaning effect of lotus leaves	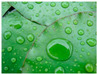	The tiny angles on the surface of the lotus leaves (e.g., caused by passing breezes) cause water balls to roll down due to gravity, carrying away the attached dust particles and cleaning the leaves without the use of detergents or the consumption of energy

**Table 4:** Improvements in the semantics of the design of the biostimulation system prototype for the lower limb rehabilitation robot.

Living Prototype	Application Area	Advantages	Disadvantages	Improvements from This Study
Bamboo fiber	Lightweight structure	Environmentally friendly, high toughness, renewable	Single morphological mapping, without combining motor function	Multi-level mapping (form + motion + environment) to improve lightweightness and durability
Arthropod joint	Multi-degree-of-freedom motion	Flexible and adaptable to complex movements	Lack of quantitative screening criteria, the design is subjective	A similarity matrix is used to screen the optimal prototype and optimize the diversity of joint motion
Lotus leaves clean themselves	Surfacing	Good anti-pollution performance, reducing maintenance costs	Not combined with rehabilitation scenario requirements	Targeted optimization of dust-proof coating materials to improve the cleaning performance of products
The hinge of the snake’s lower jaw	Joint structure	Large opening angle, strong adaptability	The matching degree with rehabilitation needs was not quantitatively analyzed	Similarity calculation verifies its adaptability and optimizes the storage and angle adjustment of the operation platform
The elm leaves fold	Flexible construction	Reserving bending space to extend service life	The specific application scenarios of rehabilitation equipment are not combined	Applied to design dust-proof parts and improve the durability and adaptability of products

## Data Availability

The raw data supporting the conclusions of this article will be made available by the authors on request.

## Appendix A

**Table A1:** Some elements of patent knowledge mining function transformation (patents from CNKI Patent Database).

Search Term: Lower Limb Rehabilitation Robot				
Order Number	Patent Publication Number	Patent Name	Applied Technology	Function Conversion
001	CN112451320A	Weight-loss support mechanism and lower limb rehabilitation robot containing it	Supporting assembly, support member, rope assembly	The resistance is reduced
002	CN109009885B	An easy-to-use exoskeleton lower limb rehabilitation robot	Drive device, movable device	Adjustable height
003	CN112386871A	A rehabilitation robot for lower limb gait rehabilitation training that is easy to move	Motor, threaded shaft	Adjustable auxiliary structure
004	CN112370305A	A lower limb rehabilitation training exoskeleton robot and its control method	Control components, drive components	Lightweight, active training
005	CN212490661U	An intelligent medical robot for lower limb rehabilitation treatment	Human sensory system	Reduced injury
006	CN212466522U	A back connection device for a lower limb rehabilitation robot	Motion blocks	Adjustable structure
007	CN212466516U	A lower limb rehabilitation robot with an ankle motion mechanism	Pole assembly, fastening assembly	Reduced damage, adjusted lightness
008	CN212466517U	An intelligent rehabilitation robot for lower limb gait training	Crossbeam and posts	The training functions are extensive
009	CN109381325B	A five-degree-of-freedom hybrid lower limb rehabilitation robot with strong continuity	Rudder gear teeth, shock-absorber spring	High flexibility in training
010	CN112294602A	A pneumatic muscle-driven lower limb rehabilitation robot	Air-operated controller	Enhanced training flexibility
011	CN212439291U	A lower limb rehabilitation robot for exoskeleton	Pavilion, turntable	High flexibility of adjustment
012	CN112274865A	On-demand adaptive control method and system for lower limb rehabilitation robot	Interactive control system	Active training
013	CN212416290U	Rehabilitation robots	Mechanical arm, drive component	High flexibility in training
014	CN212416291U	Rehabilitation robot system	Mechanical arm, drive component	High flexibility in training
015	CN212416293U	A lower limb rehabilitation training robot	Cross shaft, electric push rod	High flexibility in training
016	CN212395746U	Lower limb rehabilitation training robot deformation assembly	Lifting assembly, cushion drive	Training postures are extensive
017	CN110507322B	A quantitative state evaluation system and method based on virtual induced electromyography	Evaluation system, evaluation method	Active training, training postures are extensive
018	CN112237523A	Lower limb rehabilitation training robot	Weight-loss device, roller skates	Reduced injury
019	CN109394476B	Automatic intention recognition of brain muscle information and intelligent control method and system of upper limb	Intelligent control method, automatic identification	Enhanced control
020	CN212347108U	A lower limb rehabilitation robot and control system based on a parallel mechanism	Three degrees of freedom	Training postures are extensive
021	CN112221072A	A lower limb rehabilitation training robot	Input mechanism, adjustment mechanism	Improved accuracy
022	CN108785997B	A flexible control method for lower limb rehabilitation robot based on variable inductance	Delsys system, motion capture equipment	Enhanced control
023	CN106333830B	A lower limb rehabilitation robot walking mechanism	Crank rocker mechanism, five-bar mechanism	Training postures are extensive
024	CN212282104U	Multi-degree-of-freedom lower limb rehabilitation robot	Six degrees of freedom	Training postures are extensive
025	CN109875848B	A horizontal lower limb rehabilitation robot training mechanism and system	Drive device, turntable mechanism	Training postures are extensive
026	CN212166113U	Lower limb rehabilitation robot	Base, support rod	Training postures are extensive
027	CN112089580A	A kind of lower limb bone rehabilitation robot motion control method based on interference compensation	Dry resistance compensation	Enhanced control
028	CN112076067A	A weight reduction mechanism for a rehabilitation robot	Regulating platform, motion drive assembly	Reduced weight
029	CN112057806A	Foot pedal lower limb rehabilitation robot control system and its method	DSP control board, permanent magnet synchronous motor	Enhanced control
030	CN112043558A	A lower limb exoskeleton rehabilitation robot that combines rehabilitation training and walking assistance	Modularity	The training functions are extensive
031	CN212038162U	A lower limb rehabilitation device	Support base, adjustment assembly	Reduced weight, active training
032	CN111973409A	A kind of adaptable lower limb rehabilitation robot	Cylinder, slider	Adjustable height
033	CN111956448A	A lower limb rehabilitation robot and its kinesthetic control method	Kinesthetic control	Enhanced control
034	CN211934790U	An exoskeleton device for a lower limb rehabilitation robot	Actuator, massager	The training functions are extensive, active training and comfort improvement
035	CN111938989A	A method for evaluating the motion stability of a robot for lower limb gait rehabilitation training with a combination of rigidity and flexibility	Rope drive element	Reduced injury
036	CN111939003A	Lower limb rehabilitation robot	Walking mechanism, ceiling bracket	The training functions are extensive
037	CN211912150U	A new type of seated Chinese medicine fumigation lower limb rehabilitation robot device	Fumigation control box, rehabilitation cabin	Chinese medicine as an adjuvant therapy
038	CN111920635A	A modular spinal cord injury rehabilitation robot with multiple positions and mechanical structures	Electric push rod, modular	The training functions are extensive and can be used by multiple patients
039	CN111920654A	A self-regulating system of wearable rehabilitation walking robots	Navar	Improved adaptability
040	CN111904793A	A lower limb rehabilitation robot and control system based on a parallel mechanism	Pillar mechanism, parallel mechanism	The training functions are extensive
041	CN107273611B	A gait-planning method for a lower limb rehabilitation robot based on the characteristics of lower limb walking	Parameter modeling and gait planning	Improved adaptability
042	CN111888719A	A rehabilitation robot to assist in physical therapy	Deceleration block, balance rod	Reduced injury
043	CN111888186A	A three-degree-of-freedom bedside exoskeleton lower limb rehabilitation robot and its use method	Three degrees of freedom	The training postures are extensive and the training flexibility is high
044	CN111888193A	Multi-posture lower limb rehabilitation robot	Weight-loss device, elevator	The training functions are extensive
045	CN111821143A	Lower limb rehabilitation robot and its control method based on a semi-direct driver	Regulation device, parameterization	Self-training, training functions are extensive, and adaptability is improved
046	CN211723889U	A lower limb rehabilitation robot	Rotator	Training postures are extensive
047	CN111789739A	A lower limb rehabilitation robot ankle motion mechanism	An ankle motion mechanism	Reduced injury
048	CN111789742A	A dynamic feedback automatic lower limb rehabilitation robot	Double-axis machine, lightweight seat cushion	Train in a wide range of postures and reduce weight
049	CN111789746A	A back connection device for a lower limb rehabilitation robot	Back connection device	High training flexibility and reduced misoperation
050	CN211705248U	Lower limb rehabilitation robot	L-shaped plate and vertical rod	Low cost, family use
051	CN211675171U	A lower limb hybrid rehabilitation robot	Joint rehabilitation facility	Training functions are extensive, improving adaptability and safety
052	CN111773037A	Single lower limb rehabilitation exoskeleton device and its control method	Controller, lower limb assembly	Self-training, improved control
053	CN111773038A	A new type of lower limb rehabilitation exoskeleton robot and control method	Soft shaft transmission, spherical joint	Improved adaptability and training flexibility
054	CN111759669A	An intelligent rehabilitation robot system for upper and lower limb coordination based on electrical stimulation and its working method	Electrical stimulation and auxiliary transportation	Self-training and training functions are extensive
055	CN111759672A	A lower limb rehabilitation mirror training method based on a lower limb rehabilitation robot	Dynamic data, industrial control computer	Enhanced control
056	CN111759681A	A fixed flexible lower limb rehabilitation training robot	Separate line boxes, flexible wearables	Train a wide range of postures to improve adaptability
057	CN110464601B	A wearable biological lower limb rehabilitation robot	Joint connection device, decoupled parallel mechanism	Reduced damage and improved adaptability
058	CN211584112U	A foot mechanism of a lower limb rehabilitation robot	Foot structures	Low cost, family use
059	CN111419644B	Operation method of rehabilitation robot, rehabilitation robot, and readable storage medium	Readable storage medium	Reduced damage and improved adaptability
060	CN211560958U	A lower limb rehabilitation robot	Three degrees of freedom	Training postures are extensive
061	CN111700767A	A multi-functional rehabilitation robot training institution and method	Door frame, rope winch	Training postures are extensive, active training and adaptability are improved
062	CN211560957U	A length-adjustable structure and a lower limb rehabilitation robot	Fixed rod, adjustable rod	Simple structure
063	CN111658438A	A lower limb rehabilitation training robot	Front and rear flexion device, cross shaft	Simple structure, improved safety, training functions are extensive
064	CN111658434A	A knee hyperextension flexible exoskeleton rehabilitation robot and rehabilitation method based on pneumatic muscle	Sensory perceptual system	Improved safety, low cost
065	CN111658445A	Hip joint structure and passive gait coordination control method for lower limb rehabilitation training	Extender plate	Improved safety and simple structure
066	CN111631914A	A method for predicting the state interval response domain of a flexible cable-driven lumbar rehabilitation robot	Interval response prediction	Improved security and control
067	CN111603171A	A method and system for determining gait parameters for lower limb rehabilitation	Parameterization	Improved control and low cost
068	CN110238863B	Control method and system of lower limb rehabilitation robot based on EEG muscle signals	Electromyography, electroencephalography	Improved adaptability
069	CN111603172A	A general gait measurement method and system based on lidar	Radar surveying	Enhanced control
070	CN211382491U	A new type of Chinese medicine sit bath lower limb rehabilitation robot device	Water bath, lift	Chinese medicine as an adjuvant treatment
071	CN211383658U	A lower limb closed-chain movement rehabilitation robot	Closed chain device	Training functions are extensive and the cost is low
072	CN111588595A	An intelligent rehabilitation robot for lower limb gait training	Weight-loss agencies	Improved adaptability and training functions are extensive
073	CN111588587A	A lower limb rehabilitation robot with balanced self-weight and its application method	Balance components	Convenient operation, improved security
074	CN111568692A	A multi-degree-of-freedom lower limb rehabilitation robot	Six-degree-of-freedom mechanism	Training postures are extensive
075	CN211326602U	Lower limb rehabilitation robot	Drive sprocket	Improved adaptability and comfort
076	CN111557828A	Control method of active lower limb rehabilitation robot for stroke based on patient-side coupling	Multi-sensor information	Improved accuracy
077	CN211300956U	A horizontal lower limb rehabilitation robot	Thigh rod, slide rail	Improved adaptability
078	CN211301942U	A lower limb joint rehabilitation robot	Foot support	Active training, simulated real training
079	CN211300961U	A suspended lower limb rehabilitation training robot	Hip joint abduction driver	Training functions are extensive
080	CN2113009530	A mobile lower limb exoskeleton rehabilitation robot and its control system	Control system, data terminal	Improved accuracy and control
081	CN111544846A	A method for training and mode switching of pure intention control rehabilitation robot	EEG acquisition	Active training
082	CN211272090U	A lower limb exoskeleton rehabilitation robot	Adaptive compensation	Reduced injury
083	C111529301A	A force position hybrid control method and vertical control of a limb rehabilitation robot	Force position hybrid control	Reduced injury
084	CX2111880830	Lower limb rehabilitation exercise robot	Roller skate	The training states are extensive and the training degree is high
085	C32111938770	A wearable lower limb rehabilitation exoskeleton robot	Two-stage deceleration	The training functions are extensive
086	CN111481400A	A lower limb rehabilitation training robot	A compression device	The autonomous adjustment and adjustment functions are extensive, and the adaptability is improved
087	CN105596018B	Human motion and potential detection device and detection method based on force sensor	Six-dimensional sensor	Training postures are extensive
088	CX2110957470	A rehabilitation robot lower limb joint structure	Torque sensor, limit	Reduced injury
089	CX2110957611	Sitting joint drive lower limb rehabilitation robot	Big and small yang swing	The posture can be widely adjusted and the weight reduced
090	CN110353952B	An assistance vehicle and rehabilitation training method for lower limb rehabilitation training	Driving frame	High training flexibility, reduced misoperation
091	CX111419632A	Redundant constraint flexible cable-driven lower limb conditioning parallel rehabilitation robot and its control method	Soft cable drive device	Low cost and family use
092	C111419644A	Operation method of rehabilitation robot, rehabilitation robot, and readable storage medium	Signal feedback	The adjustment functions are extensive, improving adaptability and security
093	CXI11407601A	A lower limb rehabilitation training robot	Vibration device, step belt	Self-adjustment and control
094	cN2109629080	A seated lower limb rehabilitation robot	Bed body, lifting mechanism	Improved adaptability and high training flexibility
095	CS2109629090	A multi-posture lower limb rehabilitation robot	Bed body, lifting mechanism	The autonomous adjustment and training functions are extensive
096	X210962914	A lower limb rehabilitation robot with multiple motion modes	The middle and leg joints	Enhanced control
097	C2109629150	Rehabilitation robots	Upper and lower rehabilitation structures	Train a wide range of states and improve adaptability
098	cX111388948A	A lower limb rehabilitation training system and method under the multi-adjustment mode	Controllable motor	Reduced damage and improved adaptability
099	CN111358659A	A robot-assisted control method, system, and lower limb rehabilitation robot	Force calculation	Low cost, family use
100	cX111359161A	Lower limb rehabilitation training robot deformation components	Always adjust the moving parts	Reduced damage and improved adaptability
101	c1135965A	Parallel-drive lower limb rehabilitation training robot	Always pad the moving part	Training postures are extensive
102	c2109037600	A step training rehabilitation robot and partial degree adjustment locking mechanism	Pole, foot support	The training postures are extensive and active, and the adaptability is improved
103	CX111297629A	Rehabilitation training method for simulating climbing stairs and lower limb rehabilitation robot	Step miller	Simple structure
104	C2106737160	A new type of seated Chinese medicine water bath lower limb rehabilitation robot device	Water bath	Simple structure, improved safety Training functions are extensive
105	X111214363A	A wearable mobile robot for lower limb rehabilitation training	Perception system	Improved safety and low cost
106	C2106314690	A rehabilitation robot based on the external bone of the lower limb	Yan Zhongban	Improved safety, simple structure
107	210612481t	The room degree of the lower limb pupillary robot is adjustable	Adjustment lever	Improved security and control
108	C210108912	The inner foot mechanism based on rope motion is used for the lower limb rehabilitation robot	A contraction device	Improved control, low cost
109	CX111053674A	A flexible cable-driven blade lower limb rehabilitation robot	Rope drive device	Improved adaptability
110	N111035536A	Lower limb rehabilitation robot	Radar measurement	Enhanced control
111	C111035538A	A kind of joint module direct drive sitting and lying lower limb rehabilitation robot	Joint assembly	Chinese medicine as an adjuvant therapy
112	CX2103552990	Rehabilitation system	Operating system	Enhanced control
113	cX210301640	A counterweight lower limb rehabilitation robot	Weight-loss agencies	Improved adaptability The adjustment functions are extensive
114	cX110974621A	A foot mechanism of a lower limb rehabilitation robot	Balance components	Convenient operation, improved security
115	X110974635A	An external osteo device for a lower limb rehabilitation robot	External osteocalcification	The training functions are extensive
116	CX110993057A	Rehabilitation training system and method based on a cloud platform and lower limb rehabilitation robot	Operating system	Improved compatibility and comfort of the robot
117	C110946742A	A device and method for transferring the center of gravity of a weight-loss vehicle-assisted lower limb robot	Weight-loss equipment	Reduced weight
118	C2102052870	A four-gel external bone chondrocyte rehabilitation robot	Large and small limb rods, slides	Improved proximity
119	CX210205291t	A kind of motion-separated lower limb gait training rehabilitation robot system	Scapular support	Active adjustment
120	CX110931715A	A control system and method for realizing the coordinated motion of lower limb robot and weight-loss vehicle	Carrying a message	Reduced weight
121	CX107149539B	A lower limb rehabilitation walking robot and control method that supports omnidirectional movement	Control system, data acquisition	Enhanced control
122	X110882131A	A lower limb rehabilitation robot	EEG acquisition	Active adjustment
123	CX1070881398	A horizontal rehabilitation robot for patients with lower limb movement disorders	Adaptive compensation	Reduced injury
124	CN1108407098	An intelligent medical robot for rehabilitation treatment of lower gum	Deceleration block, balance rod	The training functions are extensive
125	N110840712A	A lower limb rehabilitation robot system based on human–computer interaction	Interactive systems	Improved travel experience
126	CX110812122A	A method and system for sitting and standing training of a lower limb rehabilitation robot	Mobile chairs, elevators	Training postures are extensive
127	CN1086069073	A mobile, well-connected, flexible drive rubber rehabilitation robot and its implementation method	Mobile device	Self-regulated practice Training functions are extensive
128	CN110743134A	A lower limb joint rehabilitation robot	Joint angle positioner	Training postures are extensive
129	CN110711115A	Hanging lower limb rehabilitation robot	Foot source motion mechanism	Reduce weight
130	CN110680676A	The mechanical leg of the lower limb rehabilitation robot	Double-axis machine, lightweight seat cushion	Train in a wide range of postures and reduce weight
131	CN110680679A	An interactive lower limb rehabilitation training system	Official department’s continuous pressing device	High training flexibility and reduced misoperation
132	CN20991656600	A rope-driven “4 + 2” lower limb rehabilitation robot	L-shaped plate, vertical rod	Training postures are extensive
133	CN110623816A	A suspended lower limb rehabilitation training robot	Joint rehabilitation facility	Training functions are extensive, adaptability is improved, and weight is reduced
134	CX110559164	The pulling system of the lower limb seat robot	Drawing machine, lower rubber assembly	Enhanced control
135	N110570916A	A method for evaluating the rehabilitation of lower limb rehabilitation robot to rehabilitate and exercise motor function	Soft shaft transmission, spherical joint	Improved adaptability Train to be flexible
136	N110517947A	A lower limb rehabilitation training robot	Electrical stimulation, auxiliary transportation	Self-training, training functions are extensive
137	CN110164601A	A wearable biological verification lower limb rehabilitation robot	Dynamic counter, industrial control computer	Enhanced control
138	C110353944A	Sitting joint drive under the condition of rehabilitation robots	Separate line boxes, flexible wearables	Train a wide range of postures and improve adaptability
139	CN110353945A	A multi-mode lower limb rehabilitation robot	Joint connection device, disassembly parallel mechanism	Reduced damage and improved adaptability
140	X110353952A	A crash prevention vehicle and rehabilitation training method for lower limb rehabilitation training	Foot structures	Low cost, family use
141	N110339023A	A lower limb rehabilitation robot that stimulates acupoints to produce reflexes	Readable storage medium	Reduced injury and improved adaptability
142	CN110327186A	Weight reduction control method, system, equipment, and storage medium for lower limb rehabilitation robot	Three degrees of freedom	Training postures are extensive
143	CN1103020338	A seated Chinese medicine water bath lower limb rehabilitation robot system	Door-type cabinet frame, rope roller	The training postures are extensive, active training, and adaptability is improved
144	CN110302034A	A kind of Chinese medicine dust bath lower limb rehabilitation robot system	Fixed rod, adjustable rod	Simple structure
145	CX110279557A	A lower limb rehabilitation robot control system and control method	Front- and rear-layer extension device, cross shaft	Simple structure, improved safety, training functions are extensive
146	N110279560A	A rehabilitation robot that uses the contralateral upper limb to control the lower limb	Perception system	Improved safety, low cost
147	CN1074119396	A single case of lower limb disability dedicated to an assistive rehabilitation robot	Extender plate	Improved safety, structural separation is easy
148	CN1066932848	A lower limb rehabilitation and adjustment medical robot with the function of transportation	Interval response prediction	Improved security, improved control
149	CN110265112A	A three-dimensional gait rehabilitation training method for a lower limb rehabilitation robot	Parameterization	Improved control and low cost
150	CN110238863A	Control method and system of lower limb rehabilitation robot based on EEG muscle signals	Electromyography, electroencephalography	Improved adaptability
151	CN110200785A	A seated Chinese medicine re-vaporization lower limb rehabilitation robot system	Measure the movement	Enhanced control
152	CN110169992	A multi-functional upper and lower glue rehabilitation robot	Remove the rehabilitation components from top to bottom	The training functions are extensive
153	CX110169893A	A lower limb rehabilitation training robot	Three degrees of freedom	The training functions are extensive and the cost is low
154	N110151496A	A multi-position lower limb rehabilitation robot and its use method	Weight-loss agencies	Improved adaptability and the function of wide migration can be adjusted
155	CN2092039560	A multi-degree-of-freedom dynamic lower limb rehabilitation robot	Balance components	Training postures are extensive
156	CK2092039610	A lower limb rehabilitation robot sugar hanging weight-loss device	Weight-loss suspension equipment	Reduced weight
157	N110037893A	A flexible cable for a dynamic wearable lower limb rehabilitation robot	Need-driven	Improved adaptability and comfort
158	N110025454	A counterweight lower limb rehabilitation robot	Multi-sensor information	Improved adaptability
159	CX110025455A	A limb exoskeleton rehabilitation robot	Thigh rod, slippery	Improved adaptability
160	CN20912279600	A lower limb rehabilitation robot for seven-degree-of-freedom movement	The feet are supporting	Active training Simulation of real training
161	CX209123286J	A full-body rehabilitation robot	Hip joint abduction driver	The training functions are extensive
162	N110013648A	A human–computer interaction system and method for a lower limb rehabilitation training robot	Control system, final data compilation	Improved accuracy and control materials
163	C2000608840	A wearable lower limb rehabilitation robot	Corrosion current collection	Active adjustment
164	CN109953761A	A lower limb rehabilitation robot perception system and motion intention reasoning method	The reasoning method	Improved accuracy
